# Correction: Welter et al. Characteristics of Nephroblastoma/Nephroblastomatosis in Children with a Clinically Reported Underlying Malformation or Cancer Predisposition Syndrome. *Cancers* 2021, *13*, 5016

**DOI:** 10.3390/cancers13225743

**Published:** 2021-11-16

**Authors:** Nils Welter, Angelo Wagner, Rhoikos Furtwängler, Patrick Melchior, Leo Kager, Christian Vokuhl, Jens-Peter Schenk, Clemens Magnus Meier, Stefan Siemer, Manfred Gessler, Norbert Graf

**Affiliations:** 1 Department of Pediatric Oncology and Hematology, Saarland University, 66421 Homburg, Germany; nils.welter@uks.eu (N.W.); s9aowagn@stud.uni-saarland.de (A.W.); rhoikos.furtwaengler@uks.eu (R.F.); 2 Department of Radiation Oncology, Saarland University, 66421 Homburg, Germany; patrick.melchior@uks.eu; 3 St. Anna Kinderspital, Department of Pediatrics, Medical University Vienna, Kinderspitalgasse 6, 1090 Vienna, Austria; leo.kager@stanna.at; 4 Section of Pediatric Pathology, University of Bonn, Venusberg-Campus 1, 53127 Bonn, Germany; Christian.vokuhl@ukbonn.de; 5 Division of Pediatric Radiology, Clinic for Diagnostic and Interventional Radiology, University of Heidelberg, Im Neuenheimer Feld 430, 69120 Heidelberg, Germany; jens-peter.schenk@med.uni-heidelberg.de; 6 Department of General, Visceral, Vascular and Pediatric Surgery, Saarland University, 66421 Homburg, Germany; clemens-magnus.meier@uks.eu; 7 Department of Urology and Pediatric Urology, Saarland University, 66421 Homburg, Germany; stefan.siemer@uks.eu; 8 Developmental Biochemistry and Comprehensive Cancer Center Mainfranken, Theodor-Boveri-Institute/Biocenter, University of Würzburg, 97074 Würzburg, Germany; gessler@biozentrum.uni-wuerzburg.de

In the original article [1] there was a mistake in Table 2 as published. Table 2 contains wrong percentages in lines *Bilateral disease* and *Patients with CPS or GU*. For this reason the table should be replaced with the correct one as shown below. 

The authors apologize for any inconvenience caused and state that the scientific conclusions are unaffected. The original article has been updated.

## Figures and Tables

**Table 2:** Association between nephroblastomatosis (NBL) and CPS or malformation in the whole cohort of patients and bilateral disease; * chi-square *p* ≤ 0.001. WT: Wilms tumor; CPS: cancer predisposition syndrome; NBL: nephroblastomatosis; GU: Genitourinary malformations; BWS: Beckwith-Wiedemann spectrum; IHH: isolated hemihypertrophy; DDS: Denys-Drash syndrome; WAGR: Wilms tumor, aniridia, genitourinary abnormalities, range of developmental delays.

	Isolated NBL		WT + NBL		WT Only		Total	
Total	73	2.5%	64	2.2%	2790	95.3%	2927	100%
Bilateral disease	31	12.3%	61	24.1%	161	63.6%	253	100%
Patients with CPS or GU	22 *	12.9% *	11	6.4%	138	80.7%	171	100%
Patients without CPS or GU	51	1.9%	53	1.9%	2652	96.2%	2756	100%
WAGR	7 *	35.0% *	2	10.0%	11	55.0%	20	100%
BWS	7 *	21.9% *	3	9.4%	22	68.8%	32	100%
IHH	5 *	17.2% *	0	0.0%	24	82.8%	29	100%
DDS	1	4.2%	2	8.3%	21	87.5%	24	100%
GU	2	3.0%	4	6.1%	60	90.9%	66	100%
