# Supplementary material for: Characteristics of Nephroblastoma/Nephroblastomatosis in Children with a Clinically Reported Underlying Malformation or Cancer Predisposition Syndrome
Source: Cancers (Basel). 2021 Oct 7;13(19):5016. doi: 10.3390/cancers13195016 (PMC8507684; doi:10.3390/cancers13195016)
Supplement: Supplementary file 1 [file cancers-13-05016-s001.zip › cancers-1353010-supplementary.pdf]

Supplementary Materials: Characteristics of Nephroblastoma/Nephroblastomatosis in Children with a Clinically Reported Underlying Malformation or Cancer Predisposition Syndrome

Nils Welter, Angelo Wagner, Rhoikos Furtwängler, Patrick Melchior, Leo Kager, Christian Vokuhl, Jens-Peter Schenk, Clemens Magnus Meier, Stefan Siemer, Manfred Gessler and Norbert Graf

Table S1. Further WT associated syndromes and malformations (number of involved patients in brackets). VSD: ventricular septal defect; ASD atrial septal defect

**Other syndromes**		Gigantism (4), Trisomy 21 (2), VACTERL (2), Perlman-syndrome (2), Neurofibromatosis type I (2), Townes-Brooks, Prader type III, Bloom-syndrome, Klinefelter syndrome, Gorlin-Goltz syndrome, Fanconi syndrome, Conradi-Hünermann syndrome, Rubinstein–Taybi syndrome, Oral-facial-digital syndrome, Mulibrey nanism, Trisomy 8, Trisomy 18, Trisomy 1, Crigler–Najjar syndrome, 11P syndrome
**Malformations**	cardiac	VSD (4), ASD (2), Fallot, Pulmonary valve stenosis, PDA (3), other cardiac defects (2)
	renal	Horseshoe kidney (5)
	facial	Auricular dysplasia (2), cleft lip and cleft palate (3), Microphthalmia, Anophthalmia, Iris coloboma, Hereditary ptosis
	visceral	anal atresia, omphalocele, lateral thyroglossal cyst, tracheal stenosis
	limb	syndactyly (2), hexadactyly, Amelia, pigeon toe, congenital hip malformation
	cerebral	isolated cerebellar atrophy
	neoplastic	lipoma (3), lymphangioma, stromal granuloma

Table S2. Data on outcome of different patient groups. (NBL: Nephroblastomatosis); *p<0,05. WT: Wilms tumor; CPS: cancer predisposition syndrome; NBL: nephroblastomatosis; GU: Genitourinary malformations; BWS: Beckwith-Wiedemann spectrum; IHH: isolated hemihypertrophy; DDS: Denys-Drash syndrome; WAGR: Wilms tumor, aniridia, genitourinary abnormalities, range of developmental delays.

	**Event *n***	**Last event (months)**	**Death *n***	**Last death (months)**	**EFS 2y**	**EFS 5y**	**EFS 10y**	**OS 2y**	**OS 5y**	**OS 10y**
No syndrome	65	115	54	143	88,2	85,3	84,1	95,0	93,0	92,2
With syndrome	16	83	15	86	87,1	84,2	83,2	96,3	92,0	88,1
**WAGR**	2	83	2	19	87,5	87,5	77,8	88,4	88,4	88,4
**GU**	7	21	7	86	87,6	87,6	87,6	96,9	92,0	88,3
**DDS**	1	5	2	6	94,7	94,7	94,7	90,6	90,6	90,6
**BWS**	8	59	3	85	83,0	**60,6***	**60,6***	100	95,5	80,4
**IHH**	4	22	2	40	84,6	84,6	84,6	100	91,8	91,8
**WT without NBL**	62	111	60	143	87,7	85,6	84,6	94,9	92,8	91,7
**Isolated NBL**	10	115	0	-	85,0	83,3	76,1	100	100	100
**WT with NBL**	14	61	4	38	80,5	71,8	69,1	94,7	92,4	92,4

Table S3: Frequencies of metastatic disease in patients with or without malformations or CPS. WT: Wilms tumor; CPS: cancer predisposition syndrome; NBL: nephroblastomatosis; GU: Genitourinary malformations; BWS: Beckwith-Wiedemann spectrum; IHH: isolated hemihypertrophy; DDS: Denys-Drash syndrome; WAGR: Wilms tumor, aniridia, genitourinary abnormalities, range of developmental delays

	**Metastasis at diagnosis**		**Non-metastatic disease**		**Total**	
**WT/NBL without CPS/malformation**	515	18.7%	2241	81.3%	2756	100%
**WAGR**	1	5.0%	19	95.0%	20	100%
**GU**	5	7.6%	61	92.4%	66	100%
**DDS**	1	4.2%	23	95.8%	24	100%
**BWS**	2	6.3%	30	93.8%	32	100%
**IHH**	5	17.2%	24	82.8%	29	100%
